# Editorial: Mental health in primary health care

**DOI:** 10.3389/fpsyg.2023.1190410

**Published:** 2023-05-17

**Authors:** Alejandra Aguilar-Latorre, Bárbara Oliván-Blázquez, Ana Porroche-Escudero, María J. Serrano-Ripoll, Rosa Magallón-Botaya

**Affiliations:** ^1^Aragonese Primary Care Research Group (GAIAP), Institute for Health Research Aragón (IIS Aragón), Zaragoza, Spain; ^2^Department of Psychology and Sociology, University of Zaragoza, Zaragoza, Spain; ^3^Research Network on Chronicity, Primary Care and Health Promotion (RICAPPS), Carlos III Health Institute, Madrid, Spain; ^4^Centre for Health Inequalities Research, Faculty of Health and Medicine, Lancaster University, Lancaster, United Kingdom; ^5^Health Research Institute of the Balearic Islands (IdISBa), Palma, Spain; ^6^Primary Care Research Unit of Mallorca, Balearic Islands Health Services, Palma, Spain; ^7^Department of Medicine, Psychiatry and Dermatology, University of Zaragoza, Zaragoza, Spain

**Keywords:** mental health, primary care, social determinants of health, depression, anxiety, psychoeducation, health inequalities

Globally, mental health disorders are on the rise, particularly during the COVID-19 pandemic. According to some studies, 25–35% of patients utilizing Primary Care (PC) services have a psychiatric condition, with more than 80% of these patients presenting depression or anxiety disorders. Many of these conditions are influenced by socioeconomic factors. General Practitioners (GPs) refer ~5–10% of psychiatric patients detected in PC to Mental Health Services, highlighting that the majority of mental disorders are diagnosed and managed in PC. Furthermore, PC rarely conducts screening and action on social determinants of health (SDH). As a result, the underlying cause of mental health issues remains unaddressed.

Given the current global situation and the available resources in PC, there is a need to investigate new approaches. Experimenting with new technologies (e.g., treatments and diagnosis) and primary prevention through a novel SDH approach that necessitates collaboration across multiple sectors and levels outside of the health system are examples of these.

The Research Topic investigated the management and prevention of mental health issues in PC. The following topics were taken into account:

- Depression and anxiety in Primary Health Care.
- Interventions and management.
- New technologies, both for treatment, and instruments for the diagnosis.
- Social determinants of mental health.
- Social DH approach to prevention and management.
- Moral injury.
- Burnout in health.

This Research Topic focused on Mental Health in Primary Health Care. The topic was published in July 2021, call for papers was published until April 2022. Scholars and practitioners were invited to submit research articles and brief reports on Mental Health in Primary Care to the journal Frontiers in Psychology. Many academics and practitioners responded to this call for papers by submitting their research. Out of a total of 24, thirteen submissions were accepted and published under this theme.

In particular, and within the topic of Depression and anxiety in Primary Health Care, we have an article that explores the factors that influence having better mental health. More specifically, Tao et al. explored whether individuals with a growth mindset might have better mental health and life event outcomes than those with a fixed mindset. Data was collected from 2,505 freshmen at a Chinese university using the Growth Mindset Scale, Adolescent Self-rating Life Events Checklist, and The Symptom Checklist-90-Revised Scale. Their findings suggested that individuals with a growth mindset are less prone to mental health problems than individuals with a fixed mindset.

On the other hand, the influence that some personality traits have on suicidal behavior has also been explored. To be more precise, Chen G. et al. aimed to test a model in which the quality of life (QOL) and suicide relationship was mediated by hopelessness and moderated by impulsivity. They used psychological autopsy data to confirm the negative association between QOL and suicide, and they demonstrated the role of hopelessness in mediating the QOL-suicide relationship, which is further modified by impulsiveness.

The relationship between mental health and cognitive impairment has also been taken into account since Zhou et al. aimed to investigate the longitudinal associations between changes in depressive symptoms and cognitive decline and mild cognitive impairment (MCI) incidence among Chinese rural elderly individuals. They concluded that among Chinese rural residents, increased depressive symptoms were associated with more cognitive decline and a higher risk of incident MCI. Along the same vein, Wang et al. identified correlates of the incidence of cognitive impairment among older Chinese populations through the use of logistic regression analysis-based decision tree approaches. They concluded that combining logistic regression and decision tree analyses can reduce predicted risk scores, allowing for the subdivision of populations with different characteristics and providing intuitive and specific insight into the effects of individual variables on predictive analyses.

Following the same topic, the use of healthcare resources and the use of psychiatric medication have been analyzed. Specifically, Lear-Claveras et al. determined the changes in pharmacological variables and variables related to the use of healthcare resources, in a population undergoing active treatment for depression or anxiety. The results indicated that anxiolytic drug use increased in comparison to the 6 months preceding the lockdown. Antidepressant use, on the other hand, was found to be decreasing. Six months after the lockdown ended, the use of health resources remained below pre-pandemic levels. The increase in drug use, particularly benzodiazepines, may indicate a worsening of symptoms during the lockdown and in the months following.

Furthermore, the psychological wellbeing of individuals can also be analyzed according to the chronic illnesses that a person suffers from. Thus, Aldossari et al. compared the psychological wellbeing of individuals who are prediabetic, diabetic, or non-diabetic. Their research showed that diabetic or prediabetic people have significantly lower psychological wellbeing than non-diabetic people. In this same line of chronic diseases, Chen H. et al. aimed to establish a nomogram prediction model to assess the occurrence of depression in patients with Systemic lupus erythematosus (SLE). Their developed nomogram provides a highly predictive assessment of depression in SLE patients, allowing for more comprehensive depression evaluation in standard clinical care. Likewise, Napalai et al. assessed the influence of COVID-19-related knowledge on mental health, healthcare behaviors, and quality of life among the elderly with non-communicable diseases (NCDs) in Northern Thailand. Their findings highlighted the significance of COVID-19-related knowledge in improving self-care behaviors and quality of life in the elderly population with NCDs during the pandemic, particularly given the high rate of stress and mental health problems in their sample.

Regarding the topic of interventions on mental health, Li et al. aimed to provide a comprehensive understanding of the trajectory, key themes, and prospects in acceptance and commitment therapy (ACT) research. Their review was the first of its kind, attempting to systematically examine the knowledge structure and map the evidence of ACT research. It expands on existing research, suggests new research directions, and makes recommendations for future ACT research.

Also, information about new instruments for the diagnosis has been added, as are the articles by Garrote-Cámara et al. and Wei et al. On the one hand, Garrote-Cámara et al. evaluated the psychometric properties of the Spanish version of the Corrigan Agitated Behavior Scale (ABS), in a sample of patients with severe mental disorders. Their results suggested that the reliability and validity of the three dimensions were acceptable. In their study, the three domains aim to explain 64.1% of the total variance of the scale, which exceeds the 50% found in the original version. On the other hand, Wei et al. aimed to test the reliability and validity of the Chinese version of the Life-threatening Illness-Family Carer Version (QOLLTI-F-CV). They concluded that the three-domain QOLLTI-F-CV questionnaire is a valid and reliable tool for identifying Quality of Life concerns among family caregivers of advanced cancer patients in China. The refactoring structure is well suited to Chinese culture and value system.

Regarding Social Determinants of Mental Health, Chela-Alvarez et al. assessed the evolution of the concern about employment status, anxiety, and depression of hotel housekeepers (HHs). They concluded that, in HHs, the COVID-19 pandemic has caused significant concern about employment status and symptoms of depression and anxiety. In the pandemic's uncertainty, variables that confer stability, such as internal locus of control, perception of social support, and a stable job, benefit mental wellbeing. Longitudinal findings indicate that the COVID-19 pandemic has long-term effects on mental health. To adequately address the anticipated influx of needs, additional resources in primary care must be allocated.

Continuing with the theme of the influence of Social Determinants of Health, Bentué-Martínez et al. explained the behavior of depression in a Mediterranean region of Northeastern Spain from an ecological and diachronic perspective. They concluded that epidemiological studies could provide useful guidelines for proactive decision-making when viewed through a territorial lens. When developing policies for creating healthier environments and directing health services with more specific resources to where they may be needed, the integration of data on diseases and territory must be considered.

All the authors developed novel research approaches and methodologies that aided theory and practice in this critical research domain of Mental Health in Primary Health Care.

Regarding the topics “Moral injury and Burnout in health” also considered, no article was received or accepted. These issues remain pending work in future Research Topics.

## Author contributions

AA-L: concept, design, and drafting of the manuscript. BO-B, AP-E, RM-B, and MS-R: critical revision of the manuscript. All authors contributed to the article and approved the submitted version.

